# An IoT Hardware Platform Architecture for Monitoring Power Grid Systems Based on Heterogeneous Multi-Sensors †

**DOI:** 10.3390/s20216082

**Published:** 2020-10-26

**Authors:** Phuoc Duc Nguyen, Hieu Quang Vo, Linh Ngoc Le, SeokJin Eo, LokWon Kim

**Affiliations:** Computer Science and Engineering Department, Kyung Hee University, Global Campus, Yongin 17104, Korea; phuocdn@khu.ac.kr (P.D.N.); 2019310178@khu.ac.kr (H.Q.V.); lnlinh93@khu.ac.kr (L.N.L.); esj@khu.ac.kr (S.E.)

**Keywords:** partial discharge detection, heterogeneous sensors, MCU, FPGA, ad hoc network

## Abstract

Partial discharge (PD) is a major indicator of various failures in power grid systems. PD exhibits a physical occurrence where a localized electrical discharge happens in insulation materials. This phenomenon causes damage to the insulating parts and, in various circumstances, leads to complete insulation breakdown. As a consequence, it can produce more costly outcomes such as abrupt outages or lost production. Therefore, PD detection plays a vital role in preventing insulation failure. In this work, we propose a system that utilizes heterogeneous sensors for the PD detection along with multi-sensor interface, real-time advanced denoise processing, flexible system operation, and Bluetooth-low-energy (BLE)-based ad hoc communication. Among the variety of heterogeneous sensors, several are developed by the application of nanomaterials and nanotechnology, thus outperforming the regular types. The proposed system successfully identifies the presence of PD from sensor signals using a microprocessor-based processing system and effectively performs an advanced denoising technique based on the wavelet transform through field-programmable-gate-array (FPGA)-based programmable logics. With the development of the system, we aim to achieve a solution with low cost, high flexibility and efficiency, and ease of deployment for the monitoring of power grid systems.

## 1. Introduction

The world nowadays has entered an era of rapid evolution owing to advancements in technology. Electricity has become an essential resource to ensure the evolution of the globe, social infrastructure, and personal daily life. Therefore, maintaining a stable and uninterrupted power supply for humanity must be a critical and crucial mission. Furthermore, ensuring the quality of insulation is of great importance because insulation materials are utilized in every phase of the power system from generation to transmission and distribution. Insulation degradation poses a challenge for the reliability as well as the continuity of power grids, and partial discharge is among the primary factors contributing to the breakdown of insulators.

The partial discharge (PD) is the initial expression in case of insulation material degradations. The phenomenon takes place when there is a breakdown in the material used for high voltage insulation [1]. It has been identified as one of the primary sources that cause material fading and insulation quality degradation. The defect stems from the impurity or cavity inside the insulation material. The PD can lead to severe insulation damages together with critical compromises in the stability of the power grid. Therefore, the study of PD is actively rising, and the detection of PD using various sensors has increasingly attracted research attention. Various instrument failures are related to PD activities in many ways. The gradual effect on electrical instruments such as insulator deteriorations and life shortages can lead to more severe problems such as large-scale power outages. Therefore, a proper and non-intrusive mechanism for the detection of PD is necessary and vital to avoid catastrophic damages to electrical equipment, saving a large amount of replacement and maintenance expenses. Furthermore, the PD detection can serve as an early warning and help to prevent unpleasant incidents in the power grid system where constant and stable electricity transmission must be guaranteed. When the PD takes place, various physical properties such as electromagnetic emissions (e.g., radio wave, light, heat), acoustic emissions (e.g., sound and ultrasonic), and chemical reactions (e.g., ozone, nitrous oxides) are manifested, and energy is subsequently released [2,3,4]. The process of PD detection measures those quantities. The approach proposed in this paper is implemented to utilize various heterogeneous sensors, including a novel piezoelectric type developed from the investigation of the associating events and with the advancements in nanomaterials. Various sensors can be attached to the proposed hardware platform with wavelet-denoise processing and ad hoc communication capability. Using heterogeneous sensors decreases the chance of missing the PD events because PD has a transient nature with a short pulse. The entire system offers a cost-effective solution along with high flexibility where power grid systems are deployed.

The article is organized as follows: Section 2 reviews different systems constructed for the power grid monitoring and the PD detection along with a denoising technique. Section 3 describes how the system is constructed with its components and depicts the mechanism of the wavelet-based denoise processing. Specifically, Section 3.1 elaborates on the characteristics of the nanotechnology-based piezoelectric sensor. Section 3.2 describes the hardware platform structure and its suitability for the implementation of the denoising accelerator based on discrete wavelet transform (DWT). Section 3.3 introduces the features of the communication module, the network structure, and its connections. Section 4 presents experiments performed on the system and discusses the obtained results as well as the advantages the system provides. Experiments were conducted to confirm the PD detection ability of the nanotechnology-based piezoelectric sensor (Section 4.1), the denoise capability of the hardware accelerator for facilitating the PD detection (Section 4.2), and the data transmission (Section 4.3). A comparison with other existing systems to highlight the benefits of our system is included in Section 4.4. The final section concludes the article with various directions for future development. Preliminary results, which proposed the heterogeneous sensors and the architecture, have been presented as a conference paper [5].

## 2. Related Works

Previously, the monitoring and examination of power grid systems have been done manually or with wired instruments, which lead to higher cost or inefficiency. The Internet of Things (IoT) era brings many benefits to those operations. Over the years, various works have been conducted to construct systems for monitoring power grid systems more efficiently. The usage of heterogeneous sensors, different computation engines, and a communication mechanism is widely applied in those systems. The authors of [6,7] proposed a Micro-controller Unit (MCU)-based device for monitoring photovoltaic (PV) power generation systems, where three types of sensors—temperature, irradiance, and humidity—were utilized to characterize the PV panels. The authors of [8] presented an MCU-based system that employed current and temperature sensors for monitoring thermal activities in power electronic converters of wind turbines. A smart sensor that incorporated FPGA for a high-speed recording of voltage and current signals was created in [9] to detect power quality disturbances. The usage of heterogeneous sensors provides systems with alternatives in case one of the sensors malfunctions, as compared to using only one type of sensor [10]. The authors of [11] summarized many types of sensors proper for insulation monitoring and PD detection, including piezoelectric, high-frequency current transformer (HFCT), Transient Earth Voltage (TEV), ultrawide band (UWB), ultraviolet (UV) light, optical sensors, etc. Most systems use only a specific type of sensor: piezoelectric [12], UV [13], or optical fiber [14]. The authors of [15] used a HFCT sensor for PD diagnosis in power cables. [16] evaluated an adapted UWB antenna to identify various features of a PD source while [17] developed an MCU-based system for measuring PD activities using broadband radiometer sensors. [18] combined a fiber network with an MCU-based control unit for monitoring PD activities on high voltage bushings. A combination of heterogeneous sensors for the identification of PD events was proposed in [19,20]. As noise inherently incorporates sensor signals, a denoising technique is essential for accurate identification of the PD event. Wavelet-based denoising proved to be effective in denoising effect with fast calculation speed not only in PD detection [21,22,23] but also in other application fields [24,25,26]. Furthermore, the wavelet-based denoising also acts as an useful pre-processing step to extract meaningful features before feeding into artificial neural networks for more advanced analyses such as disturbance classification, fault recognition [27,28]. In this paper, an IoT hardware platform architecture for monitoring the power grid system is proposed. It implements a hardware accelerator that performs wavelet-based denoising to facilitate the PD detection, and it is able to interact with heterogeneous sensors and present a BLE-based ad hoc network for transferring data. A nanotechnology-based piezoelectric sensor, specifically designed to react to PD events, is also introduced. Heterogeneous sensors can complement each other toward the anomaly detection, such as missing events with small-magnitude pulses. Various sensors help increase the number of features for the detection task, reduce the detection threshold, and perform a more precise diagnosis. The use of the BLE-based ad hoc networks not only assists the data transmission with modest power consumption but also eliminates bulky wiring in contrast to wired systems. The hardware platform (Xilinx Zynq-7000 All Programmable System-on-Chip [29]) (Xilinx, San Jose, CA, USA) utilizes the processing system as a central unit for controlling tasks. At the same time, it can offload high-speed processing and complex works (e.g., signal denoising) on programmable logic (FPGA), providing a higher level of freedom than the case of using only a microcontroller unit (MCU) [30,31].

## 3. System Design

**Figure 1:**
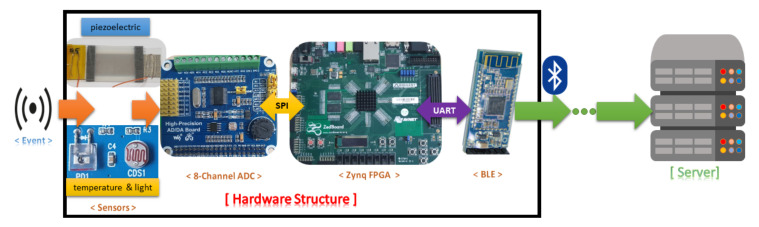
System block diagram.

The main components of the system include three parts (aside from the remote server): the sensors and the signal interface, the IoT hardware system platform, and the communication module, as indicated in Figure 1. The use of heterogeneous sensors helps cover extensive details of the phenomenon of interest, lowering the chance of false alarm or missing the event. The sensor part can interface with the main processing unit through a multi-channel analog-digital converter (ADC) module (Waveshare Electronics, Shenzhen, Guangdong, China). Thus, multiple sensors can have a standard interface to the development board through a peripheral connection such as the serial peripheral interface. The hardware platform, including both MCU and FPGA, provides flexibility and effectiveness for both controlling and processing tasks. The BLE module (JNHuaMao Technology Company, Jinan, Shandong, China) brings a low-cost communication solution to the system. The subsequent subsections describe each system component in more detail.

### 3.1. Nanotechnology-Based Piezoelectric Sensor

A sensor device takes a particular stimulus from changes in the physical properties of the surroundings and produces responses to the fluctuations. Combined with the nanotechnology, which is a field of science that performs research to manipulate atoms and molecules in the nanometer scale, nanotechnology-enabled sensors facilitate the detection solution with enhanced sensitivity and improved accuracy [32]. Relying solely on data from one sensor may compromise the sensitivity due to environmental noises. Therefore, the proposed system utilizes multi-heterogeneous sensors: a nanotechnology-based piezoelectric sensor, a temperature sensor, and a light-dependent resistor. The novel piezoelectric sensor introduced in this article utilizes an array of zinc oxide (ZnO) nanorods. Nanorods are one form of nanostructures that have a dimension of less than 100 nm. ZnO is widely recognized as an excellent candidate to develop the desired sensor. The material possesses excellent semiconducting, piezoelectric, and optical properties useful for constructing electromechanical devices, optical devices, etc. [33]. Therefore, ZnO has been chosen for developing our piezoelectric sensor. ZnO exhibits an abundance of 1D nanostructures such as nanorods, nanotubes, nanowires, etc., which add many desirable attributes such as transparency, large-area fabrication, and large bandgap. The opening of the bandgap improves the response to the generated photo-excited free electrons caused by UV light energy. Thus, with the development currently in progress, another sensor type (ultraviolet) will soon be introduced and added to the sensor heterogeneity in the system.

**Figure 2:**
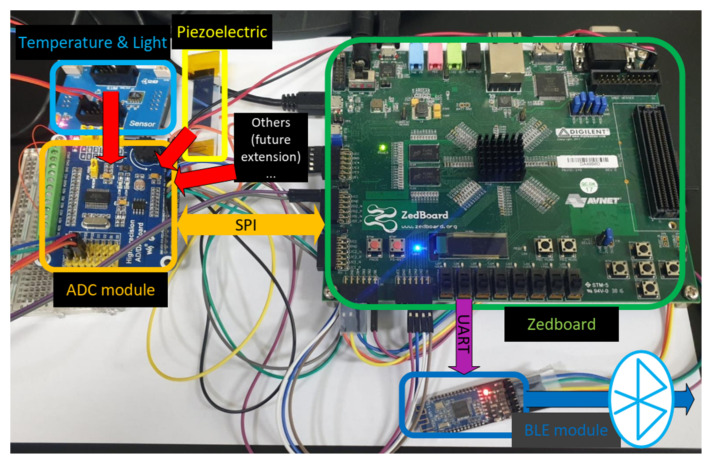
The proposed hardware platform.

The piezoelectric effect is a phenomenon that happens inside semiconductors such as ZnO. The effect stems from the ability of the crystalline structure to generate an electrical current in response to an applied mechanical stress [34]. Acoustic emission creates a wave to propagate the surface of piezoelectric material, which in turn converts mechanical motions into an electrical signal. With proper placement in the power transmission network, the PD event can be detected effectively and also be identified for the location of occurrence using the arrival time of acoustic waves from multiple sensors at various positions [35,36]. Different algorithms to find the acoustic wave arrival time were proposed by [37,38]. By applying nanomaterials and nanotechnology, the piezoelectric effect based nanogenerators have been explored as self-powered sensors. A novel ZnO nanorod-based nanogenerator developed by a sensor team of Kim et al. [39] can be employed as a piezoelectric sensor to detect the PD through converting vibrations (acoustic or ultrasonic waves) into an electrical voltage. With the versatility and diverse configurations on nanostructures, ZnO is a favorable piezoelectric material. ZnO has a reliable response to the piezoelectric effect owing to the crystal structure where Zn${}^{2+}$ and O${}^{2-}$ ions stacked alternatively along the *c*-axis, and the center is the place where positive and negative charges cancel each other. Furthermore, ZnO with a wurtzite crystal stands out among various popular piezoelectric materials such as lead zirconate titanate (PZT), polyvinylidene difluoride (PVDF), molybdenum disulphide (MoS_2_) due to non-toxicity, low cost, and ease of fabrication [40,41]. With a vertical arrangement, the distribution of ZnO nanorods is dense and uniform, which facilitates the piezoelectric effect. The constructed piezoelectric sensor has been tested under a controlled PD generation, and it produced a maximum voltage of approximately 40 mV, sufficient for use in a digital system through the multi-channel ADC module. The proposed hardware platform with accompanying sensors is shown in Figure 2.

### 3.2. Hardware Platform and Denoise Accelerator

This part presents the detail of the hardware platform and the signal processing mechanism. It also explains how the platform is suitable for our purpose as well as elaborates on the denoising algorithm.

#### 3.2.1. Hardware Platform Overview

The heterogeneous sensor system is built around the Zedboard [42] (Avnet, Phoenix, AZ, USA), which features a Xilinx Zynq-7000 All-Programmable System-on-Chip (AP SoC) device for high-speed and advanced processing operations and ease of control. The Zedboard is an evaluation and development kit with plentiful interfaces to enable a wide variety of applications such as video processing, motor control, function acceleration, and general prototyping. It combines a processing system and Xilinx programmable logic into a single device. The device features the Xilinx XC7Z020-1CLG484C Zynq-7000 AP SoC (Xilinx, San Jose, CA, USA) with a dual-core ARM Cortex-A9 CPU at heart (ARM, Cambridge, UK), 512 MB DDR3 RAM (Micron, Boise, ID, USA), and 256 Mb QSPI Flash (Cypress, San Jose, CA, USA). The programmable logic is equivalent to Artix-7 FPGA in Xilinx 7 Series, with resource availability summarized in Table 1.

**Figure 3:**
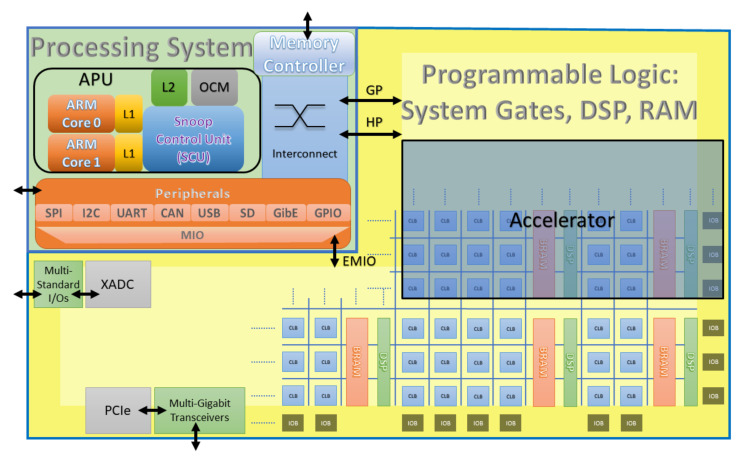
Components of the main processing unit.

The created system is aiming towards flexibility and effectiveness for both controlling and signal processing tasks. Using solely MCU can cope well with controlling but may fail on high-speed data processing. FPGA, on the other hand, can deal effectively with high-speed processing operations based on its logic part but is not easily adjustable after the logic is identified. The Zynq-7000 AP SoC hardware platform provides both MCU operation and FPGA logics at a modest cost. Figure 3 shows the components in the Zynq-7000 AP So C with two main parts: processing system (PS) and programmable logic (PL). The PS provides a quick prototyping environment for schedule or control tasks, while the FPGA-based PL gives the flexibility to implement custom hardware designs to sample and process the data. The PS is considered as MCU with an ARM-based processor and an ample amount of built-in peripherals such as the serial peripheral interface (SPI), inter-integrated circuit (I2C), controller area network (CAN), universal asynchronous receiver-transmitter (UART), general purpose input/output (GPIO), etc., which is ideal for interacting with sensors. The PS is responsible for detecting the point when PD happens, which results from the crossing over a certain level of sensor signals. The PL, based on FPGA with the flexibility in implementation, can perform various types of hardware acceleration, such as offloading tasks that require real-time processing services from the PS and reclaiming the processor bandwidth. The PL implements a signal processing mechanism (i.e., wavelet denoising) to make the detection more accurate and noise resistant. The PL is advantageous to the system since it helps to separate the processor from heavy computations of the denoise signal processing.

#### 3.2.2. Denoise Processing

**Figure 4:**
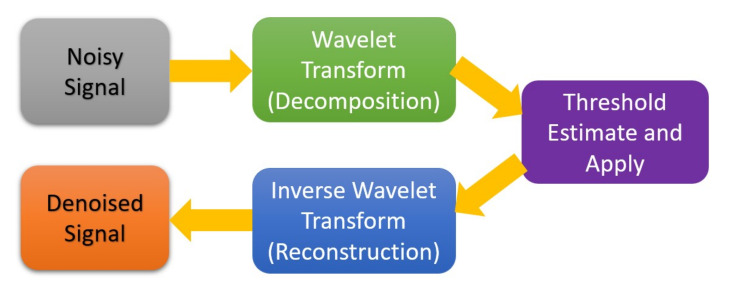
Basic steps in wavelet denoising.

**Figure 5:**
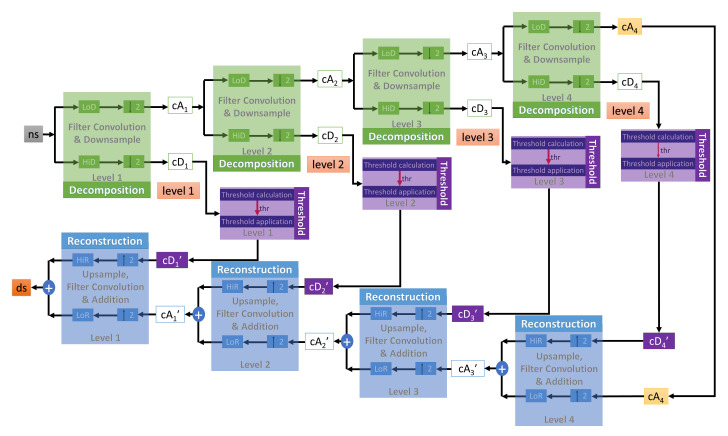
Detail operations in each denoising step with a total of four levels.

A denoise technique is implemented as a hardware accelerator in the PL to facilitate the real-time operation of the system. Among 28 denoising techniques such as fast-Fourier-transform-based, lowpass filtering, Wigner–Ville-distribution-based, recursive least squares, and exponentially weighted recursive least squares methods, a wavelet transform-based denoise has demonstrated to give the best performance [43,44]. Thus, it has been implemented for real-time noise removals in our system. The wavelet transform, which results in shifted and scaled signals with regard to a mother wavelet, is expressed by the following equation:

(1) $$\Psi_{f}^{\psi }\left(\tau ,s\right)=\frac{1}{\sqrt{\left|s\right|}}\int_{-\infty }^{+\infty }f\left(x\right)\psi^{*}\left(\frac{x-\tau }{s}\right)dx$$

where τ = translation parameter, s = scale parameter, ψ(*x*) = mother wavelet. The processing mechanism in this work is based on a fast algorithm for wavelet transform developed by Mallat [45], which produces two groups of coefficients: approximation (*cA*) and detail (*cD*). As shown in Figure 4, the noisy signal needs to go through three phases of processing for noise removal: decomposition, thresholding, and reconstruction. The decomposition is based on the wavelet transform, while the reconstruction is the result of the inverse operation. The working details of each step can be observed in Figure 5 with four levels of denoising.

**Figure 6:**
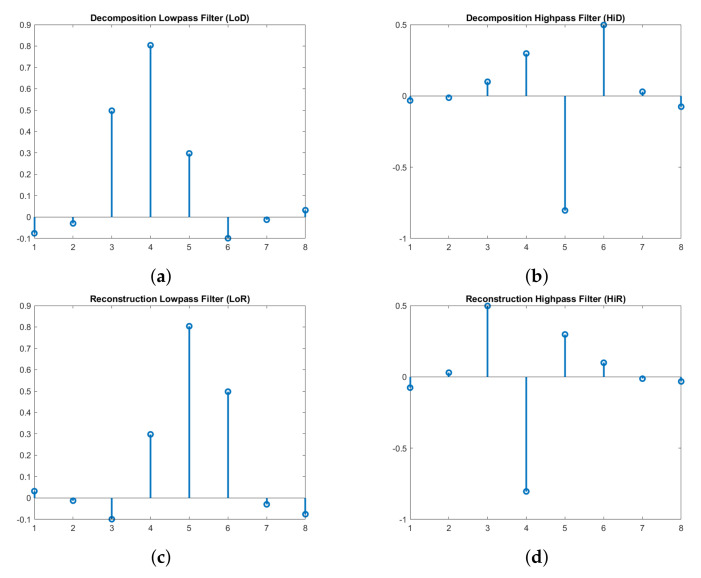
Four filters of the symlet4 wavelet: (**a**) Low-pass in Decomposition (LoD). (**b**) High-pass in Decomposition (HiD). (**c**) Low-pass in Reconstruction (LoR). (**d**) High-pass in Reconstruction (HiR).

The approximation and detail coefficients are the result of a convolution operation between the input signals with low-pass (LP) filters and high-pass (HP) filters of a wavelet, respectively. The filters in the reconstruction stage are reversed in terms of time, compared to the ones used in the decomposition stage [46], as illustrated in Figure 6.

The wavelet transform, when applied to various real-world signals, results in a sparse representation and thus settles the signal expressions into a small number of wavelet coefficients with large magnitudes. A shrinkage operation truncates some coefficients with small magnitudes. However, the truncated coefficients do not affect the overall quality of the signal since they contribute as noise components. The shrinkage operation takes place when a threshold value is applied to every detail coefficient at each level by hard or soft threshold functions, given as the following equations:

(2) $$D^{H}\left(y,t\right)=\left\{\begin{array}{c} y\;\left|y\right|\geq t\\ 0\;\left|y\right|<t \end{array}\right.$$

(3) $$D^{S}\left(y,t\right)=\left\{\begin{array}{c} sign\left(y\right)\left(\left|y\right|-t\right)\;\left|y\right|\geq t\\ \;\;0\;\;\;\;\;\left|y\right|<t \end{array}\right.$$

Because the hard threshold function creates some discontinuities and oscillations, the soft threshold is used to create a smooth signal transition [47]. The threshold estimation at level *i* (denoted as $\lambda_{i}$) where there are a total of $n_{i}$ detail coefficients with the median $m_{i}$ is calculated from the universal thresholding estimator [48]:

(4) $$\lambda_{i}=m_{i}/0.6745\cdot \sqrt{2\cdot log(n_{i})}$$

Signal processing with orthogonal wavelets such as Symlet or Daubechies produces the most compact signal representation [49]. Therefore, the Symlet [50,51] with four vanishing moments (symlet4) is used as the mother wavelet in our denoising accelerator. The small number of saved coefficients after the decomposition step in the transformation makes the operation storage efficient. The signal after reconstruction is more apparent and sharper because noise components have been erased. In the experimental section, the denoising operation has first been implemented in software for fast development, and then a hardware model is developed for verification.

### 3.3. Communication Module

**Figure 7:**
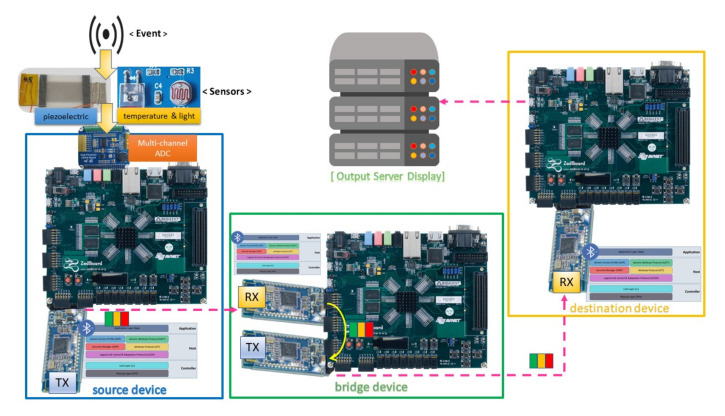
Network structure and connection.

The system is equipped with a BLE connection to form an ad hoc network for flexible deployment and data transmission. In addition to obtaining the sensor data, the PS performs wireless data transmitting through an HM-10 BLE module [52].The wireless data transfer is necessary because it helps eliminate bulky and cumbersome wiring. The low power consumption of the BLE contributes to prolonged lifetime usage [53,54]. The BLE module interacts with the development board through a UART connection. It aids in forming an ad hoc network where communication is performed in a peer-to-peer manner, which facilitates system deployment. The module is preferred over a built-in communication mechanism onboard such as Ethernet because of its low power consumption and flexibility. Although the BLE technology has the limitation on the data buffer size (around several hundreds KB of RAM), throughput (due to processing delay), and transmission range (around tens of meters) [55,56,57], it is sufficient for our targeted application. Additionally, as reliability is an important factor in sensor systems, [58] proposed a method of excluding poor channels to ensure reliability and maintain a minimal package loss in BLE-based sensor systems. The BLE operates in a 2.4 GHz Industrial, Scientific, and Medical (ISM) band containing 40 channels with 2 MHz spacing apart. Bluetooth communication is utilized in accordance with the aforementioned development platform to demonstrate the feasibility of a communication-capable PD detection system. In the setup, there are three types of devices: source device, bridge device, and destination device (Figure 7). Among the utilized BLE modules, the module which initiates the connection is a master (e.g., the TX in the source and bridge device) while the other one is a slave (e.g., the RX in the bridge and destination device). The slave module is in the state waiting for the connection. The master module establishes a connection with the slave module by sending the MAC address of the slave. The source device is devised attached to one novel and two regular sensors through the ADC board with an SPI interface; therefore, the source device can easily obtain data from the sensors. In order to transmit the data wirelessly, the source device is equipped with an HM-10 BLE module, which eliminates the bulky wires for transferring important data. The bridge device is an intermediate one, which acts as a link to connect the source device with the destination device. The bridge device will receive the information from the source device and forward the information to the intended recipient of the information, i.e., the destination device. The usage of the bridge device helps to close the gap in distance between the source device and the destination device. The bridge device is equipped with two HM-10 BLE modules: one is for receiving data, and the other one is for transmitting data. With this configuration, each transmission module solely focuses on its specified task without overlapping functionality, saving the bandwidth for its predetermined function (transmit/receive) with the cost of double hardware (two BLE modules are utilized on the bridge device). The destination device is the designated receiver of the information sent by the source device. The data goes through the source device, passes through the bridge device, and reaches the destination device. It is also possible for the destination device to send the data through the Internet to get into a remote server for data monitoring and more in-depth processing.

## 4. Experiments and Results

This section describes various experiments conducted on the proposed system in PD detection, denoise processing, and data transmission. It also points out the advantages to which the proposed system brings in comparison with other types.

### 4.1. PD Detection from the Piezoelectric Sensor

**Figure 8:**
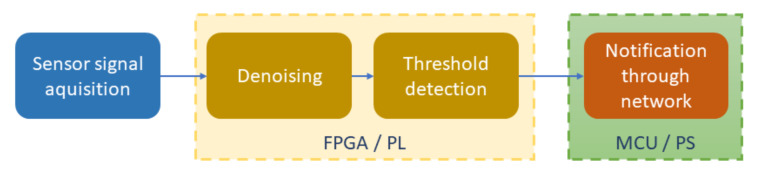
Data flow of the PD detection process.

Since PD events occur under extreme situations and require a huge size research equipment and environment to incur the events artificially, the research in this paper utilized sensor signals captured under PD occurrences and provided by the sensor research teams collaborating in a joint-project that Korea Electric Power Corporation (KEPCO) leads. The signals have been recorded as data in memory (DDR) and played back to the system in our overall experimental environment. The sensor has been experimented under the laboratory environment of the sensor teams where the PD was generated to test the reactions of the sensor to the event. When the PD happened, the voltage of the piezoelectric sensor was recorded to be about 30 mV. The sensor performed with satisfactory sensitivity and adequate accuracy, but the produced signals had small magnitudes. Furthermore, the signals might easily be affected by various noise sources such as electromagnetic noise, power electronic components, switching [59]. The combined effect of noises from multiple sources was manifested as white noises to the sensor signal. Although applying white noises might not completely reflect the complicated and unpredictable real-world conditions, it is supportive in illustrating the effect of interference because of its random distribution in time and frequency domains [60,61]. The PD detection process, combined with denoising and notification steps, is illustrated in Figure 8. After acquiring the sensor signal, the FPGA-based denoising accelerator will perform the noise removal to make the signal cleaner, facilitating the thresholding step for the PD detection. We implemented a dedicated hardware block to compare the denoised sensor signal with a threshold value configured by the software in the MCU. The output of the hardware block is connected to MCU as an interrupt signal. Once the threshold is higher than the previously configured threshold value, the interrupt signal connected to the MCU would be triggered. After the interrupt trigger, the MCU goes to an interrupt service routine to send the notification message to a server through the ad hoc network.

### 4.2. Implementation of the Denoising Architecture and Results

**Figure 9:**
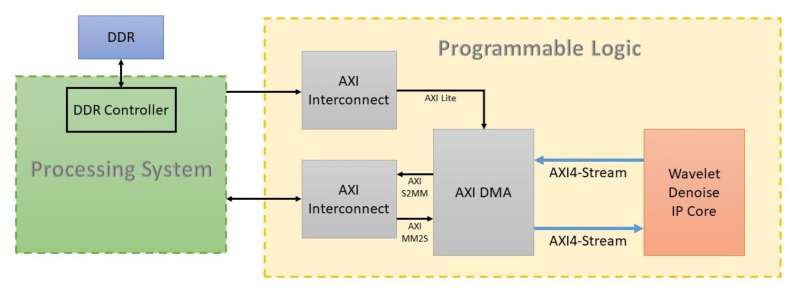
Wavelet Denoise IP core within the hardware platform.

**Figure 10:**
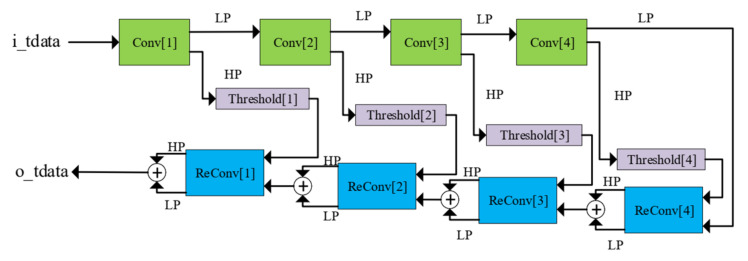
Coarse view of the Denoise IP core.

The wavelet denoise core inside the hardware platform is depicted in Figure 9, along with various advanced extensible interface (AXI) connections [62]. The DMA core provides a convenient method of configuration through AXI4-Lite to deliver high-speed data from memory to the wavelet-based denoise accelerator. The AXI stream is the main interface of the wavelet core to the AXI DMA (Xilinx, San Jose, CA, USA) [63], ensuring a dedicated, point-to-point, and efficient data transfer with no addressing context required. The wavelet core is designed to accommodate the real-time operation and high-speed processing with its coarse view (Figure 10), including Conv (Convolution), Threshold, and ReConv (Reconstruct-Convolution).

**Figure 11:**
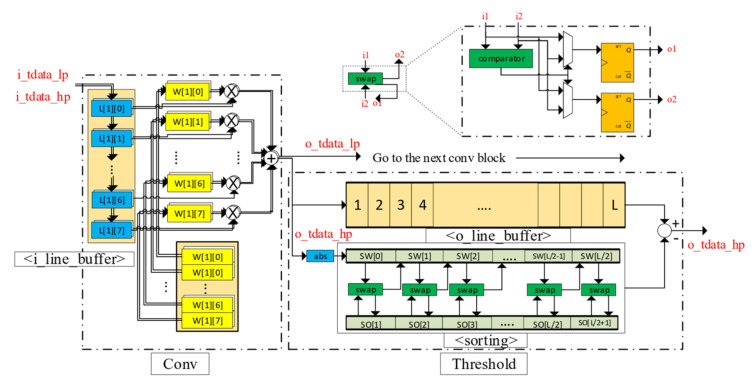
Fine-grain view of the two fundamental blocks: Conv and Threshold.

The Conv and ReConv have the same structure and components (line buffers, weight memories, multipliers, adders, etc.). Their differences lie in the purpose of the loaded weights: Conv for Decomposition (analysis) and ReConv for Reconstruction (synthesis). Each convolution part is preloaded with filter weights of the symlet4 and has dedicated resources (multipliers, adders) for calculation, as shown in the left part of Figure 11. Line buffers are windows to perform the convolution operation and have the same length as the weight memory, i.e., *n* = 8 is the number of elements of each filter for symlet4. As each downsampled input signal is shifted into the line buffer, the convolution result is produced as the summation of multiplications between each weight element and the corresponding value in the line buffer, which is expressed by the following:

(5) $$\begin{array}{c} o\_tdata\_*=\sum_{j=1}^{n}L\left[i\right]\left[j\right]\times W\left[i\right]\left[j\right]\\ \mathrm{where}\;i=0\;\mathrm{for}\;\mathrm{LP}\;\mathrm{and}\;i=1\;\mathrm{for}\;\mathrm{HP} \end{array}$$

The downsampling operation keeps every other result from the convolution module and is performed before data enters the Conv block. The action of discarding half of the total data samples not only reduces the amount of data but also eliminates the effect of aliasing when the data are restored in the reconstruction step. The threshold step is crucial to the denoising purpose. It dynamically determines a threshold value on the data stream, which in turn is applied to the result of the HP convolution path to obtain the detail coefficients at a particular level. The threshold module consists of two buffers named sorted (*so*) and swapped (*sw*) and a sorting module, as in the right part of Figure 11. The number of elements in each buffer is half the length of the data entering the threshold module, which are stored on another line buffer to be truncated after the threshold value is determined. With new data shifted in from the Conv block at every clock cycle, each element in the sorted buffer is compared with the previous element in the swapped buffer, and their contents are updated as in Algorithm 1.
**Algorithm 1:** The sorting algorithm on so and sw buffers.
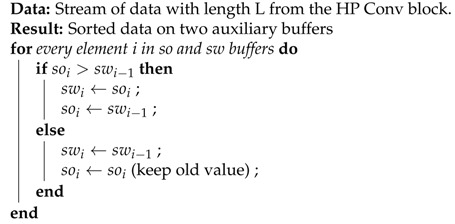


**Figure 12:**
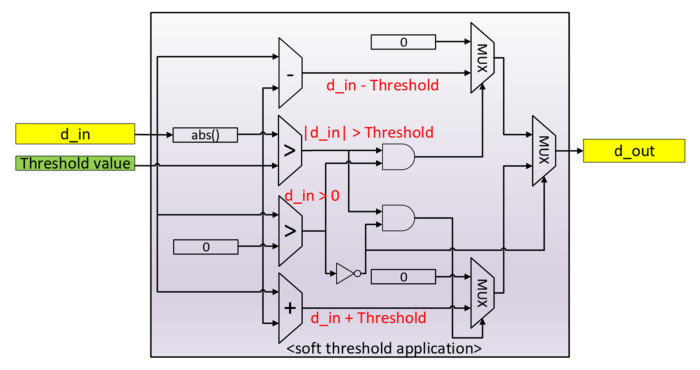
Soft threshold application circuit.

The sorting algorithm ensures that the median value of the data stream is pushed towards the higher end (larger index) of the two auxiliary buffers. This median value is then used to calculate the threshold value for one level, and the threshold application block will apply a soft threshold, as shown in Figure 12.

**Figure 13:**
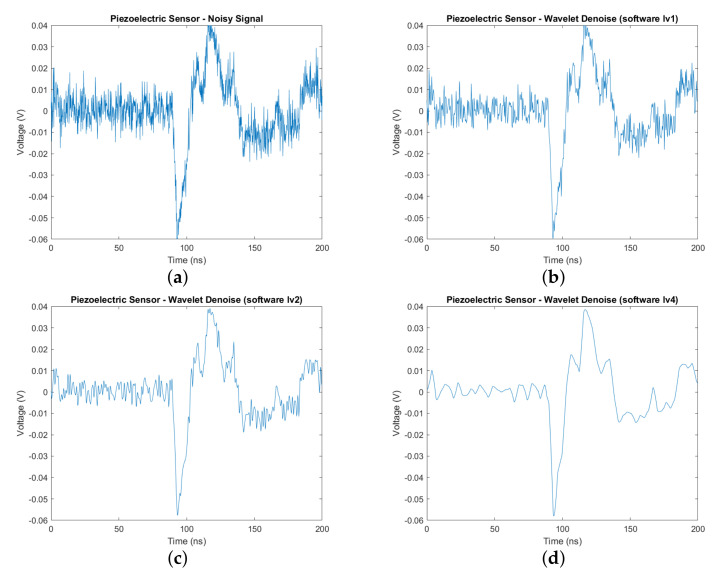
Wavelet Denoise with various levels using software implementation: (**a**) Noisy signal. (**b**) Wavelet denoise level 1—software version. (**c**) Wavelet denoise level 2—software version. (**d**) Wavelet denoise level 4—software version.

**Figure 14:**
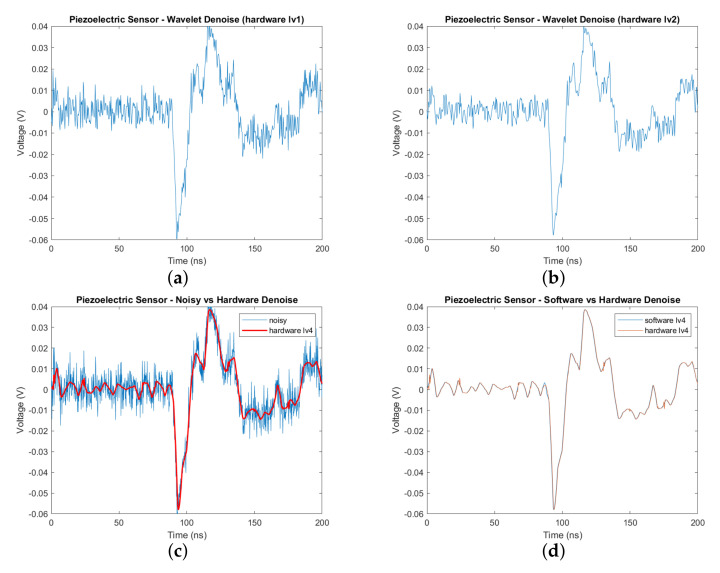
Wavelet Denoise with various levels using hardware implementation: (**a**) Wavelet denoise level 1—hardware version. (**b**) Wavelet denoise level 2—hardware version. (**c**) Wavelet denoise level 4 (hardware) vs noisy signal. (**d**) Wavelet denoise level 4—hardware vs software.

**Figure 15:**
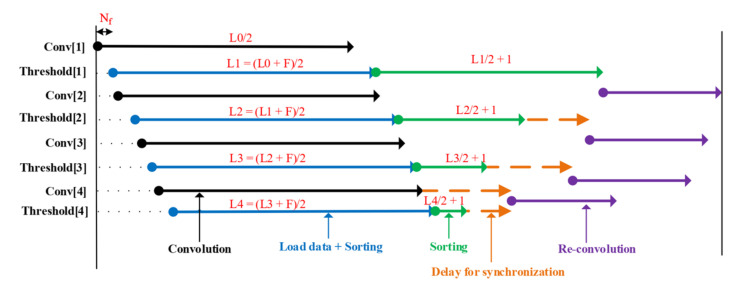
Timing diagram of Denoise IP core.

Figure 13 shows the software implementation result of wavelet denoising with different levels of decomposition: one, two, and four. It particularly indicates that the more levels applied, the clearer the sensor signal is. With level 4 denoising, we observe that there is little noise in the signal, and the peaks in the signal are easily identified as PD occurrences. The proposed architecture can accommodate various levels of choice as well as perform a fast computation for each stage in the process. The result from software implementation was saved and used to compare with the data obtained from hardware implementation to verify the results. As shown in Figure 14, the differences in two types of implementation at every denoising level were very insignificant and nearly imperceptible by bare eyes (Figure 14a,b). As seen from Figure 14c, the hardware-obtained data were much smoother and easier to identify the PD than the original, noisy input data. The denoise data of the hardware implementation at level 4 were also plotted against the software version in Figure 14d. The two data representations mostly overlapped, with small magnitude spikes in the hardware due to a slight loss of accuracy during the data quantization process. The hardware design used a signed 16-bit fixed-point data type (Q0.15), while the software version utilized a 64-bit floating-point data type. Table 2 indicated high values of correlation coefficients between the two implementations, further justifying the correctness of the hardware accelerator. Therefore, the obtained results proved the correctness as well as the effectiveness of the hardware version of wavelet-based denoising. The design was successfully synthesized and could run at 150 MHz in FPGA using the hardware resource shown in Table 3. The denoise IP took 1774 cycles or 11.84 μs for the stream of 1001 sensor data to complete its operation. Figure 15 represented the timing information with all activities: convolution, data loading, sorting, and data synchronization. $N_{f}$ = 1 was the initial loading time into the Conv block while, *L0* = 1001 and *F* = 8 were the length of the sensor data and the total filter elements, respectively. After sorting to find the threshold value, delay cycles were inserted to synchronize the data for feeding into the re-convolution operation, which requires two separate sets of computed coefficients: detail (*cD’*) and approximation (*cA’*). Because of downsampling at each level, the convolution time was nearly the same across four levels while the latency of the denoise core did not change with the increase of denoise levels. The first level had the highest number of elements for convolution and sort, thus incurring the most operation time while the downstream levels (level 2, 3, and 4) were running. Therefore, the proposed hardware architecture provided the advantage of giving better signals without introducing more time.

### 4.3. Ad Hoc Networking

**Figure 16:**
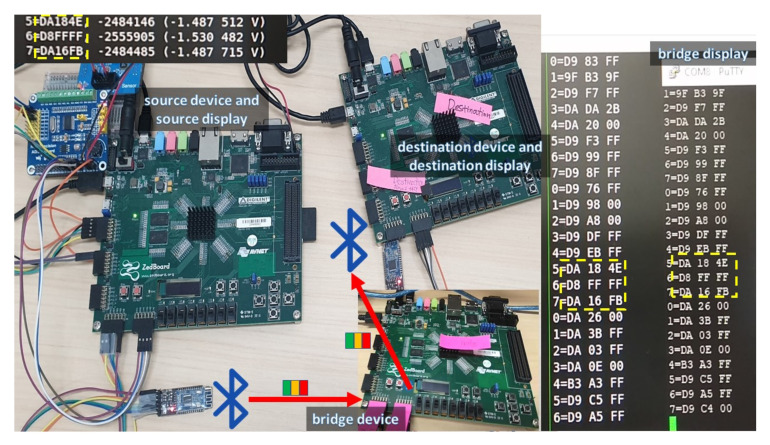
Result of data transmission.

Another experiment was conducted to test the feasibility of the system for signal interfacing and data transmission. Because of the highly specialized equipment required for the PD generation of the piezoelectric sensor, multi-sensor interfacing was verified through the multi-channel ADC with two supporting sensors: temperature and light. One Zedboard was utilized as the source device and connected to the HM-10 module so that the denoised data could be originated from the source and transmitted through a bridge node before the data reached the destination. The data has been successfully transmitted through the BLE modules and retrieved at the target node. In our experiment, in order to verify the transmission correctness, the data was displayed and compared on a terminal of the destination device, as shown in Figure 16. The integration of routing network protocols and error checking code [64,65] will be examined in the future work to address the scalability and increase adaptability to faulty data and losses.

### 4.4. Comparison with Existing Systems

Table 4 presents other systems for PD applications. None of them uses heterogeneous sensors for covering various details of the phenomenon of interest, thus limiting their detection capability. Their sole computation platform makes them possible for only simple control without noise removal and requires another powerful computing machine for complex processing. The lack of a mechanism for wireless data transfer also makes them cumbersome in deployment.

In terms of denoise processing, our proposed architecture excels when compared with previous implementations (Table 5). An implementation of only wavelet transform and its inverse operation with no threshold calculation restricts its meaningful applications such as denoising. Single-level threshold value could not cover all noise in the whole signal range. By adjusting the number of storage elements, our architecture has the ability to accommodate flexible lengths of the input stream, not limited to a maximum of 32 data samples. In the statistical viewpoint, the median value used for threshold calculation reflects the signal characteristics more correctly compared to using the mean value. Thus, our characterized structure ensures a continuous, real-time, and effective denoising of the utilized sensor signals.

## 5. Conclusions

In this article, we present a highly flexible and feasible system for the PD detection of power grid systems. The proposed system can work with heterogeneous sensors, including a newly developed piezoelectric sensor specialized for detecting the presence of PD events. The piezoelectric sensor is fabricated using nanotechnology advancements with satisfactory performance in the detection outcome. In addition to interaction with the novel sensor, the proposed system, which is based on the combination of MCU and FPGA, is easy to deploy because of its capability to form an ad hoc network. The environment of power grid systems is numerous, and the sensor systems are sensitive to the environment because of various noise sources. The research proposed an advanced denoising technique in a hardware design for real-time processing and highly accurate signal quality. The programmable logic provided by FPGA technology brings more flexibility to the system when it requires to offload computation extensive processing tasks such as denoising. The system functions as a sensor node to feed data to central servers for more complex data inspection and even machine learning techniques. The proposed system aids in forecasting imminent insulation failures as well as in the determination of item replacement.

## Figures and Tables

**Table 1:** Zedboard device feature summary [29].

XC7Z020-1CLG484C Zynq-7000 AP SoC			
**Processing System**		**Programmable Logic**	
Processor Core	Dual-core ARM Cortex-A9	Xilinx 7 Series PL Equivalent	Artix-7 FPGA
Maximum Frequency	667 MHz	PL Cells	85K
L1 Cache	32 KB Instruction, 32 KB Data	Look-Up Tables (LUTs)	53,200
L2 Cache	512 KB	Flip-Flops (FFs)	106,400
On-chip Memory	256 KB	DSP Slices	220
Memory Support	DDR3 (Double Data Rate), DDR3L (DDR Low Voltage), DDR2, LPDDR2 (Low Power DDR), 2x Quad-SPI, NAND, NOR	Block RAM (# of 36-Kb Blocks)	4.9 Mb (140)

**Table 2:** Software–hardware implementation similarity.

Denoise Level	Correlation Coefs
1	0.999881
2	0.997799
4	0.994126

**Table 3:** System hardware resource @150 MHz.

Resource	Quantity Used
LUTs	31,179
FFs	55,072
DSP	64
BRAM	0

**Table 4:** Comparison with previous systems for PD applications.

Paper	Platform	Sensor	Communication	Processing	Compared to Ours
Miao 2012 [66]	Software on Personal Computer	Radio Frequency (RF) antenna	to PC	PD localization	Single type of platform (software application) and sensor (RF antenna), no data transmission
Pei 2015 [67]	FPGA	Ultra High Frequency (UHF)	local	peak identification	Single type of platform (FPGA) and sensor (UHF), local communication only
Wei 2019 [68]	MCU	piezoelectric	local	data storage and transmission	Single type of platform (MCU) and sensor (piezoelectric), local communication only
Ours	🗸 (MCU+FPGA)	🗸 (piezoelectric)	🗸 (local+global)	🗸 (PD detection and signal denoise)	

**Table 5:** Comparison with DWT implementations.

Paper	Compared to Ours
Bahoura 2010 [69]	Implement only wavelet transform and its inverse operation, no threshold operation
Bahoura 2010 [70]	Apply only one threshold value to all three denoising levels
Chen 2015 [71]	Use mean to calculate threshold value, limited number of data samples for denoise processing (maximum of 32 samples)
